# Integrative transcriptomics and single-cell transcriptomics analyses reveal potential biomarkers and mechanisms of action in papillary thyroid carcinoma

**DOI:** 10.3389/fgene.2025.1536198

**Published:** 2025-05-30

**Authors:** Wanchen Cao, Kai Gao, Yi Zhao

**Affiliations:** School of Traditional Chinese Medicine, Beijing University of Chinese Medicine, Beijing, China

**Keywords:** papillary thyroid carcinoma, weighted gene co-expression network analysis, gene set enrichment analysis, machine learning, ScRNA-seq

## Abstract

**Objective:**

Papillary thyroid carcinoma (PTC) has a high recurrence rate and lacks reliable diagnostic biomarkers. This study aims to identify robust transcriptomic biomarkers for PTC diagnosis through integrative bioinformatics approaches and elucidate the cellular mechanisms underlying PTC pathogenesis at single-cell resolution.

**Methods:**

Based on the Gene Expression Omnibus (GEO) database, we downloaded PTC-related RNA-seq datasets (GSE3467, GSE3678, GSE33630, GSE65144, and GSE82208) and an scRNA-seq dataset (GSE191288). Among these, the RNA-seq dataset GSE3467 was used as the training dataset to perform differential gene expression analysis, GO and KEGG enrichment analyses, weighted gene co-expression network analysis (WGCNA), machine learning, ROC analysis, nomogram analysis, and GSEA for mining potential biomarkers. The remaining RNA-seq datasets (GSE3678, GSE33630, GSE65144, and GSE82208) were used as the validation datasets to validate these potential biomarkers. Based on the results from potential biomarker mining, the scRNA-seq dataset (GSE191288) was used to analyze and uncover key cell types and their mechanisms involved in the occurrence and development of PTC.

**Results:**

This study retrieved relevant PTC datasets from the GEO database and identified three biomarkers (ENTPD1, SERPINA1, and TACSTD2) through a series of bioinformatics analyses. GSEA suggested that these biomarkers may be involved in the occurrence and development of PTC by collectively regulating the cytokine–cytokine receptor interaction pathways. scRNA-seq analysis revealed tissue stem cells, epithelial cells, and smooth muscle cells as key cell types in PTC. Cell–cell communication analysis revealed that epithelial cells primarily interact with tissue stem cells and smooth muscle cells through two ligand–receptor pairs, namely, COL4A1–CD4 and COL4A2–CD4. The collagen signaling pathway was identified as the most dominant pathway, and violin plots demonstrated that ligands COL4A1 and COL4A2 were highly expressed in epithelial cells, while the receptor CD4 showed elevated expression in both tissue stem cells and smooth muscle cells. Pseudotime analysis demonstrated that these three cell types underwent three distinct differentiation stages, during which the expression levels of the biomarkers ENTPD1, SERPINA1, and TACSTD2 showed stage-specific trends.

**Conclusion:**

In summary, this study combines RNA-seq and scRNA-seq analysis techniques to identify ENTPD1, SERPINA1, and TACSTD2 as potential biomarkers for PTC at the transcriptomic level and tissue stem cells, epithelial cells, and smooth muscle cells as key cells in PTC at the cellular level. This study conducted in-depth research and analysis on these potential biomarkers and key cells, providing new research foundations and insights for future basic experimental research and the diagnosis and treatment of PTC in clinical settings.

## Introduction

Thyroid cancer is a type of cancer that occurs in the thyroid gland, which is located in the neck and is responsible for producing hormones that regulate the body’s metabolism. Thyroid cancer is the most common endocrine malignancy worldwide, including papillary thyroid carcinoma (PTC), medullary thyroid carcinoma (MTC), anaplastic thyroid carcinoma (ATC), and follicular thyroid carcinoma (FTC) (Niu et al., 2017). In recent years, the incidence of PTC has increased, making it the most common type of thyroid cancer, accounting for approximately 70% of all cases (Li et al., 2018). Currently, there is still no consensus on the mechanisms underlying the occurrence and progression of PTC. Although PTC can usually be cured through surgery and radioactive iodine therapy, the high heterogeneity in clinical and pathological characteristics makes it difficult to develop effective treatment methods applicable to all patients (Ruan et al., 2023). Therefore, identifying the key genes and cells involved in the pathogenesis of PTC is of great significance for improving the clinical diagnostic level of PTC and the application of precision medicine (Pan et al., 2024).

Machine learning algorithms (Luan et al., 2023) and weighted gene co-expression network analysis (WGCNA) (Wang et al., 2023) are widely used to identify potential biomarkers for various diseases. Single-cell transcriptome analysis can provide a more precise understanding of the transcriptome within individual cells, elucidating their roles in cellular functions and how gene expression promotes beneficial or detrimental states, thereby clarifying unknown tumor characteristics that traditional bulk transcriptomic studies cannot discern (Hwang et al., 2018). Existing studies have shown that at the cellular level, PTC is composed of functionally distinct non-immune cell subpopulations and different clusters of immune cells that interact and affect clinical outcomes (Pu et al., 2021a). Studies have also shown that, 42 oncogenic signaling pathways and a six-gene panel predicted the prognosis of PTC and were associated with the tumor immune microenvironment (Wang et al., 2022). Therefore, actively exploring the molecular mechanisms of PTC will be the foundation for early identification and intervention of the disease.

Specifically, this study addresses three key questions: what are the most reliable transcriptomic biomarkers for PTC diagnosis? Which cell types and cellular interactions drive PTC progression? How do the identified biomarkers function in the tumor microenvironment at the single-cell level?

**FIGURE 1:**
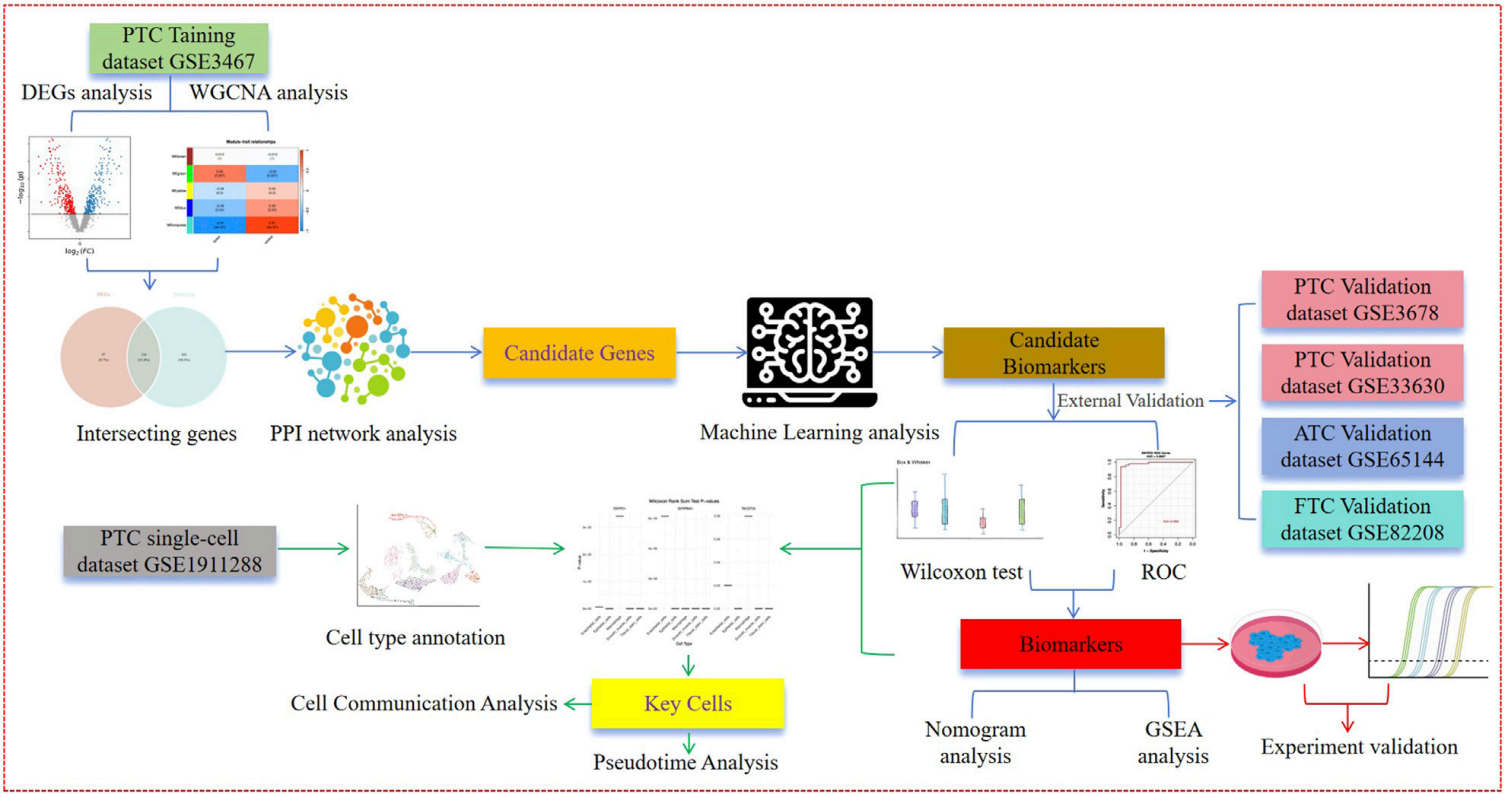
Workflow of the study.

In this study, we aim to conduct a series of bioinformatics analyses at the transcriptome and cellular levels using the GSE3467, GSE3678, GSE33630, and GSE191288 datasets to screen for potential biomarkers and key cells in PTC and analyze the regulatory mechanisms of these genes and cells. Additionally, we further validate and compare the predictive power of potential PTC biomarkers for other types of thyroid cancer, such as ATC and FTC, in the GSE65144 and GSE82208 datasets. We believe that this research provides theoretical support for further in-depth exploration of the pathogenesis and molecular mechanisms of PTC, thereby aiding in the improvement of diagnosis and prognosis for patients with these diseases. The integrative approach is illustrated in Figure 1.

## Materials and methods

### Collection and processing of datasets

PTC RNA-seq datasets and the scRNA-seq dataset were obtained from the Gene Expression Omnibus (GEO) database (https://www.ncbi.nlm.nih.gov/geo/). The PTC RNA-seq dataset with the GSE number GSE3467 was downloaded from the GEO database and used as the training set data. The PTC RNA-seq datasets with the GSE numbers GSE3678 and GSE33630, the ATC RNA-seq dataset with the GSE number GSE65144, and the FTC RNA-seq dataset with the GSE number GSE82208 were downloaded from the GEO database and used as the validation dataset. The PTC scRNA-seq dataset with the GSE number GSE191288 was also downloaded from the GEO database. A total of 203 RNA-seq datasets were included in this study: nine tumor and nine normal datasets from GSE3467, seven tumor and seven normal datasets from GSE3678, 49 tumor and 45 normal datasets from GSE33630, 12 tumor and 13 normal datasets from GSE65144, and 27 tumor and 25 follicular thyroid adenomas (FTA) datasets from GSE82208. A total of seven scRNA-seq datasets were included: six tumor and one normal dataset from GSE191288.

### Differential gene expression and GO and KEGG enrichment analyses

In order to identify the differentially expressed genes (DEGs) between the tumor and normal groups in the training set GSE3467, this study used the “limma” package to obtain DEGs from the training set. The threshold for screening DEGs in PTC was set as adjusted *p*-value <0.05 and |log2FC| > 1. DEGs were visualized using volcano plots and heatmaps created with the “ggplot2” and “ComplexHeatmap” packages. To analyze the potential biological functions and pathways that DEGs may play in PTC, GO and KEGG enrichment analyses were conducted using the R package “clusterProfiler” with a *p*-value threshold of < 0.05.

The R package used for GO and KEGG enrichment analyses is clusterProfiler, version 4.10.1, available on GitHub (https://github.com/YuLab-SMU/clusterProfiler).

### Weighted gene co-expression network analysis and identification of candidate genes

WGCNA is a sophisticated systems biology method used to elucidate the correlation patterns between genes in microarray samples. This method helps identify gene sets with strong co-expression and reveals potential candidate biomarker genes or therapeutic targets by exploring the intrinsic connections within the genome and their correlations with phenotypes. In this study, WGCNA was performed on the training set GSE3467 data, and hierarchical clustering analysis was conducted using the “gplots” software package. To ensure that gene interactions conform as closely as possible to a scale-free distribution, the optimal soft-threshold (β) was selected based on R2 (R2 = 0.85) using the “pickSoftThreshold” function; to construct gene modules, adjacency between genes was calculated, similarity between genes was computed based on adjacency, and then a similarity coefficient between genes was derived, leading to a system clustering tree of genes. According to the criteria of the mixed dynamic tree-cutting algorithm, the minimum number of genes per gene module (minModuleSize) was set to 100, and mergeCutHeight was set to 0.4 (60% similarity) to merge similar modules identified using the dynamic tree-cutting algorithm, resulting in gene modules; the R package “psych” was used to perform Pearson correlation analysis between gene modules and tumor and normal groups, with modules having |r| > 0.4 and P < 0.05 considered key modules. Furthermore, gene significance (GS) and module membership (MM) were calculated for key modules, and a scatter plot of MM and GS correlations was drawn. Hub genes were obtained with the selection criteria of MM > 0.8 and GS > 0.6. The intersection of DEGs and hub genes was obtained using the R package “VennDiagram,” yielding intersecting genes. A protein–protein interaction (PPI) network for the intersecting genes was constructed in the STRING database (http://www.string-db.org/), and finally, core genes were identified using “Cytoscape” software and its MCODE plugin, which are considered candidate genes for PTC.

The relevant computational codes for WGCNA algorithms can be accessed via the following GitHub repository: https://github.com/SamBuckberry/RUN-WGCNA.

### Machine learning algorithms to screen candidate biomarkers

To systematically screen for candidate biomarkers with significant contributions to PTC, this study used three complementary machine learning algorithms for feature selection on the training set GSE3467: the least absolute shrinkage and selection operator (LASSO), support vector machine recursive feature elimination (SVM-RFE), and the Boruta algorithm. These three methods evaluate the importance of gene features from different perspectives (LASSO: linear model based on coefficient shrinkage; SVM-RFE: nonlinear model based on support vector weights; and Boruta: random forest-based feature importance) to comprehensively assess gene signatures. To ensure the reproducibility of these algorithms, a random seed of 12,345 was set.

In the LASSO analysis, we used the “glmnet” package to construct a logistic regression model, setting the “family” parameter to “binomial” to suit the classification task. Genes with non-zero coefficients were retained as important features. The optimal regularization parameter λ was determined through 10-fold cross-validation, with lambda. min (the value yielding the minimum cross-validated error) was selected as the final model parameter. Model performance was evaluated through cross-validation curves and coefficient shrinkage plots.

For the SVM-RFE analysis, implemented using the “caret” package, features with lower contributions were sequentially eliminated through recursive feature elimination. The model training also used 10-fold cross-validation, repeated 10 times to reduce randomness, and evaluated all possible feature subset sizes. The feature subset with the lowest error and highest accuracy in cross-validation was selected as the optimal result.

The Boruta analysis was performed using the “Boruta” package, which is based on random forest construction. By comparing the importance of the original features with randomly generated shadow features, the algorithm identifies truly relevant features—retaining those with significantly higher importance than the shadow features and eliminating the insignificant features. The analysis was set to 500 iterations to ensure stability, with all default parameters tested and confirmed to be suitable for the current dataset.

Finally, these three algorithms with distinct principles demonstrate complementary advantages to some extent. The intersection of results from the three methods was analyzed using Venn diagrams and visualized as a network using Cytoscape. The intersecting genes were considered candidate biomarkers for PTC. This multi-algorithm consensus approach reduces bias inherent to any single method and enhances the reliability of the results.

The relevant computational codes for machine learning algorithms can be accessed using the following GitHub repository: https://github.com/xliu93/machine_learning_algorithms.

### Analysis of the expression of candidate biomarkers

The Wilcoxon test was used to analyze the expression of candidate biomarkers in the training set GSE3467 and validation sets GSE3678 and GSE33630 (p < 0.05). Genes that show consistent expression trends and significant differences between groups in these three datasets will continue to be considered candidate biomarkers for PTC. Additionally, the expression of candidate biomarkers in the validation sets GSE65144 and GSE82208, which represent different types of thyroid cancer, was analyzed.

The relevant computational codes for the Wilcoxon test can be accessed using the following GitHub repository: https://github.com/stenver/wilcoxon-test.

### ROC analysis of candidate biomarkers

To assess the predictive power of candidate biomarkers, the R package “pROC” was used to plot the ROC curves for each candidate biomarker and combinations of three candidate biomarkers in the sample data from the training set GSE3467 and validation sets GSE3678, GSE33630, GSE65144, and GSE82208. Candidate biomarkers with AUC values greater than 0.7 in both the training set GSE3467 and validation sets GSE3678 and GSE33630 were defined as biomarkers for PTC. Additionally, the predictive power of candidate biomarkers in the validation sets GSE65144 and GSE82208, which represent different types of thyroid cancer, was analyzed.

The relevant computational codes for ROC analysis can be accessed using the following GitHub repository: https://github.com/kb22/ML-Performance-Evaluation.

### Construction of nomograms and assessment of biomarkers

To further evaluate the predictive power of the biomarkers, nomogram models were constructed based on the biomarkers in all samples from the training set GSE3467 using the R package “rms.” Calibration curves were plotted using the R package “Rregplot” to assess the predictive accuracy of the nomograms.

The relevant computational codes for the construction of nomograms can be accessed using the following GitHub repository: https://github.com/ClevelandClinicQHS/QHScrnomo.

### GSEA of biomarkers

To further explore the biological processes in which biomarkers participate in PTC, differential analysis was performed for each gene’s high- and low-expression groups in the training set GSE3467 using the R package “limma.” Based on the differential analysis results, all genes were sorted by log2FC from high to low, obtaining the lists of related genes for the high- and low-expression groups of each biomarker. The KEGG background gene set (c2. cp.kegg.v7.4. symbols.gmt) was downloaded from the Molecular Signatures Database (http://www.gsea-msigdb.org/gsea/downloads.sp), and GSEA was conducted using the R package “clusterProfiler” to explore the potential functions of the biomarkers. To further understand the common biological pathways in which biomarkers participate in PTC, the top five pathways enriched by the biomarkers were intersected using the R package “VennDiagram.” Additionally, to analyze the enrichment of related genes (genes in the pathways) in the pathways associated with the biomarkers, the relationships of the related genes in the above-obtained three common pathways were analyzed using “Cytoscape.”

The relevant computational codes for GSEA can be accessed using the following GitHub repository: (https://github.com/junjunlab/GseaVis).

### Quality control of single-cell RNA-seq datasets

To perform quality control on the GSE1911288 PTC single-cell dataset and filter eligible cells and genes, this study first imported the raw count data h5 files using the Read10X_h5 function from the Seurat package. Subsequently, the Create Seurat Object function was applied to each dataset to create Seurat objects, which then generated gene expression matrices. The percent.mt function was used to calculate the expression proportion of mitochondrial genes in each cell. Before proceeding with further analysis, the data underwent quality control to remove low-quality cells that did not meet the criteria. Specifically, the criteria for defining low-quality cells in this study included the following: 1) the number of feature genes (nFeature) being less than 500 or exceeding 10,000; 2) the expression proportion of mitochondrial genes exceeding 30%; and 3) the count number (nCount) being below 1000 or above 10,000.

### Identification of highly variable genes

Gene expression fluctuations among different cells can result from both technical errors and biological heterogeneity. Therefore, it is essential to identify highly variable genes caused by biological levels to reduce the interference of information from other less relevant genes. Harmony is used to integrate the filtered single-cell sequencing data, and LogNormalize is applied for data standardization. The FindVariableFeatures function with the vst method is used to extract genes with large coefficients of variation between cells, and the top 2000 highly variable genes with significant fluctuations are displayed.

### Cell dimensionality reduction and cluster annotation

To observe whether the overall distribution of cells in each sample is consistent and check for any obvious outlier samples, the ScaleData function is used to scale the single-cell sequencing data. The JackStrawPlot function is used to determine the principal components with statistical significance, and the top 50 principal components with statistical significance from principal component analysis (PCA) are selected for subsequent analysis, with the top 15 results visualized. The Seurat package is used to identify small cell clusters through FindNeighbors and FindClusters, and the ClusTree function is used to visualize the interactions between different clusters at different resolutions. Cell-type annotation is crucial for scRNA-seq data analysis. This study uses the SingleR package (Aran et al., 2019), which annotates scRNA-seq data based on known cell-type marker genes. It assigns cell-type labels to individual cells based on the reference samples, using the highest Spearman rank correlation, focusing only on marker genes between labeled pairs to highlight cell-type-specific differences.

### Identification of key cells

To explore the differences in the abundance of various cell types in different samples, the abundance of each cell type in the two groups of samples is analyzed. The Wilcoxon rank-sum test is used to compare the differences in the abundance of each cell type between the two groups. Based on the results, the Wilcoxon rank-sum test is further used to compare the expression levels of biomarkers in cell types with significant changes. Cells that show significant differences in biomarker expression are defined as key cells (*p* < 0.01).

### Key cell communication analysis

To understand the interactions between key cells, the expressions of receptors and ligands are used to infer interactions between different cell clusters. The R package “CellChat” is used to analyze cell–cell communication among key cells by calculating the aggregated communication network and displaying the quantity and intensity of the interactions. Bubble charts are used to visualize the interactions between ligand–receptor pairs of cells, and heatmaps are used to visualize the signaling pathways that contribute the most to the output or input signals of key cells. Finally, violin plots are used to display the expression of representative genes on the signaling pathways with the greatest contribution.

### Pseudotime analysis of key cells

To understand the differentiation process of key cells and the expression of biomarkers at different stages of key cell differentiation, each key cell is arranged along the corresponding cell trajectory. Pseudotime analysis of key cells is completed by dividing key cells into different differentiation states based on gene expression profiles. The “Monocle2” package is used to build pseudotime trajectories for the selected cell populations. At the same time, branched expression analysis modeling (BEAM) in the R package “Monocle” is used to visualize the expression of biomarkers at different time points in the differentiation trajectory of key cells.

The relevant computational codes for single-cell RNA-seq analysis can be accessed using the following GitHub repository: https://github.com/seandavi/awesome-single-cell.

### Cell culture

The PTC cell lines (TPC-1 and IHH4) were maintained in RPMI 1640 (Gibco) supplemented with 10% FBS and penicillin (100 U/mL)/streptomycin (100 mg/mL) and cultured in a 37°C incubator with 5% CO_2_.

### Quantitative real-time PCR

Total RNA was extracted from cells using TRIzol reagent (Invitrogen), according to the manufacturer’s instructions. Quantitative real-time PCR (RT-qPCR) was carried out using the SYBR Green Mix (Vazyme). GAPDH served as a control, and the 2^^−ΔΔCt^ method was used to evaluate the expression level of each gene. The primers were designed as shown in Table 1. The statistical significance of differences between experimental groups was determined using the Student’s t-test. A two-tailed p-value of less than 0.05 (*p* < 0.05) was considered statistically significant.

**TABLE 1:** RT-qPCR primers.

Gene name	Primer	序列(5′–3′)
ENTPD1	Forward Reverse	CAGCCTTGGGAGGAGATAAA GAGAGAGGTGTGGACAATGGTT
SERPINA1	Forward Reverse	GGAGGGTCTCTGCTTTGTTT GACTAGGGAGGAGAAGGGATATAG
TACSTD2	Forward Reverse	TCCACTTGTATCATGGCCTACC CTCAAAGACATCCAAACTGCGT
GAPDH	Forward Reverse	GGTCGGTGTGAACGGATTTG GGAGTCATACTGGAACATGTAG

## Results

### Identification of DEGs and results of GO and KEGG enrichment analyses

Based on the methods described above, the DEGs between the tumor and normal groups in the training set GSE3467 were analyzed (Supplementary Table S1), resulting in 413 significantly differentially expressed genes. Among them, there were 228 upregulated genes and 185 downregulated genes in the tumor group samples compared to those of the normal group samples (Supplementary Table S2). To understand the distribution of DEGs as a whole, a volcano plot was drawn to visualize the DEGs, and the top 10 upregulated and top 10 downregulated DEGs were marked on the volcano plot (Supplementary Figure S1A). Furthermore, to display the expression levels of DEGs, this study selected the top 25 upregulated genes and the top 25 downregulated genes based on the log2FC fold change, totaling 50 genes as representatives, and drew the expression heatmap of DEGs (Supplementary Figure S1B). GO and KEGG enrichment analyses were also performed on the DEGs (*P* < 0.05). A total of 384 GO pathways (Supplementary Table S3) and 12 KEGG pathways were found to be enriched (Supplementary Table S4). Among them, there were 280 biological process (BP) pathways, 60 cellular component (CC) pathways, and 44 molecular function (MF) pathways. The top 10 pathways were visualized, with the GO enrichment analysis results shown in Supplementary Figure S1C and the KEGG enrichment results shown in Supplementary Figure S1D. In the GO enrichment analysis results, BP pathways closely related to PTC included the hormone metabolic process, thyroid hormone metabolic process, and thyroid hormone generation. In the KEGG enrichment analysis results revealed pathways closely related to PTC, including those related to the immune system, specific cancer types, the endocrine system, and energy metabolism.

### WGCNA and the identification of candidate genes based on the methods described

Based on the methods described above, a co-expression gene module was constructed for the training set GSE3467 data. The analysis results showed that there was one outlier sample in the tumor group, which was removed (Supplementary Figure S2A). The optimal soft-threshold (β = 7) was selected (Supplementary Figure S2B); a total of six gene modules were obtained (Supplementary Figure S2C); among them, the MEturquoise module had the strongest correlation with the tumor group (R = −0.91, *P* < 0.001), containing a total of 2,403 genes (Supplementary Figure S2D). Therefore, the turquoise module was considered the key module. Furthermore, GS and MM within the turquoise module were calculated, and a scatter plot of MM and GS correlations was drawn. Using the screening criteria of MM > 0.8 and GS > 0.6, 898 genes that met the conditions were identified as hub genes (Supplementary Figure S2E) (Supplementary Table S5). The intersection of DEGs and hub genes resulted in 316 intersecting genes, as shown in the Venn diagram (Supplementary Figure S2F) (Supplementary Table S6). To further understand the interactions among the candidate genes at the protein level, the candidate genes were uploaded to the STRING database for PPI network construction (interaction score >0.4), resulting in 308 nodes and 447 edges (Supplementary Figure S2G). Using “Cytoscape” software and its MCODE plugin, a total of 12 core genes were identified, which were considered candidate genes for PTC for further analysis (Supplementary Figure S2H) (Supplementary Table S7).

Having identified candidate genes through differential expression and co-expression analyses, we next applied complementary machine learning approaches to rigorously select the most robust biomarkers.

### Results of machine learning for screening candidate biomarkers

**FIGURE 2:**
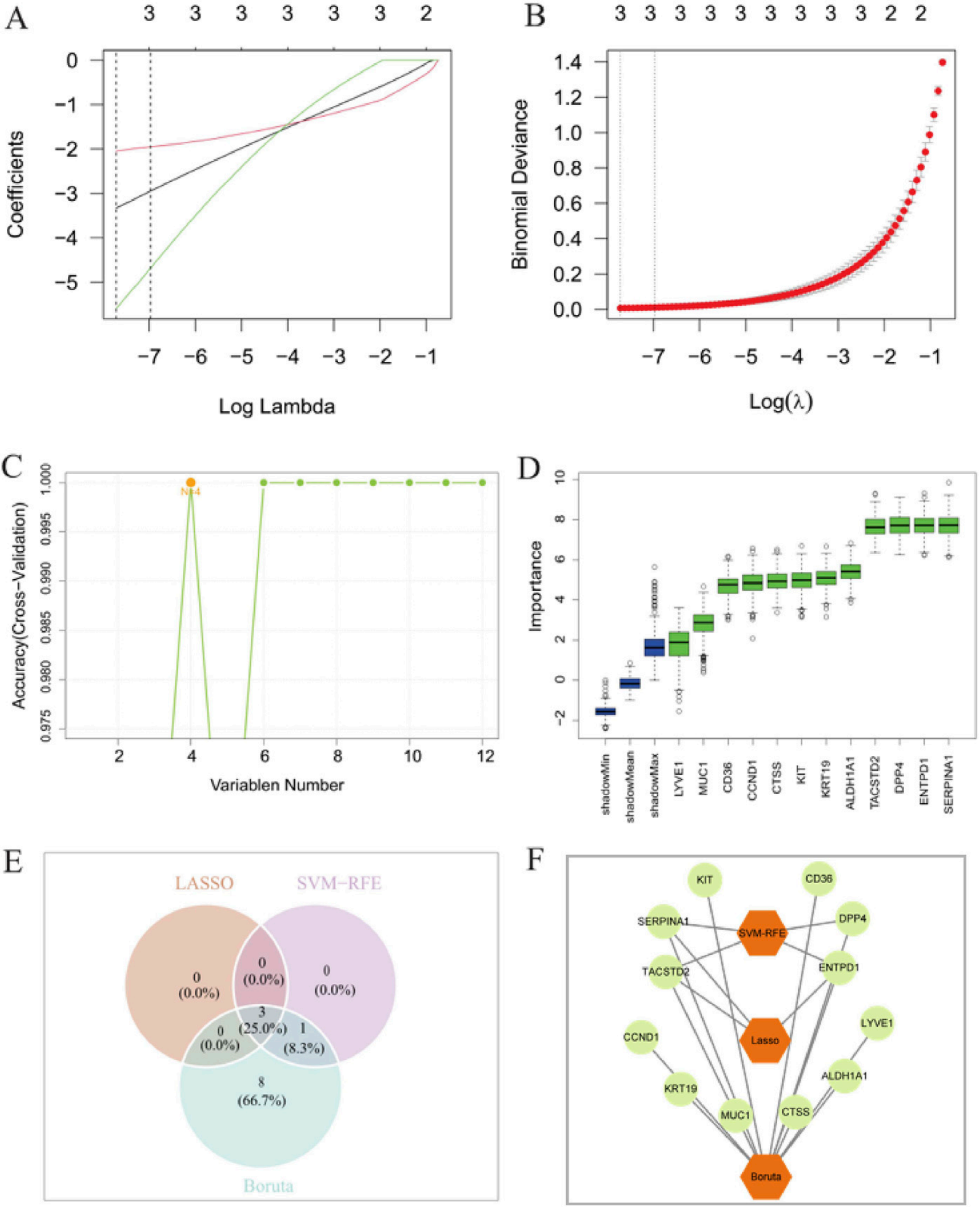
**(A,B)** LASSO algorithm; **(C)** SVM-RFE algorithm; **(D)** Boruta algorithm; **(E)** identification of candidate biomarkers; **(F)** network visualization of candidate biomarkers.

Based on the 12 PTC candidate genes selected using the aforementioned methods, LASSO analysis, SVM-RFE, and Boruta analysis were performed on the training set GSE3467 to screen for relevant feature genes. Using the LASSO algorithm, in the tumor group, the log (lambda.min) of LASSO was −7.7174, and the log(lambda.1se) was −6.97313, identifying three genes with non-zero coefficients (Figures 2A,B). The SVM-RFE algorithm identified the four genes with the highest accuracy (Figure 2C). The Boruta algorithm screened out 12 important feature genes (Figure 2D). Finally, through Venn analysis, the three genes, namely, ENTPD1, SERPINA1, and TACSTD2, were identified as candidate biomarkers for predicting PTC (Figure 2E), and “Cytoscape” software was used to visualize the results in a network (Figure 2F).

### Analysis results of the expression of candidate biomarkers

**FIGURE 3:**
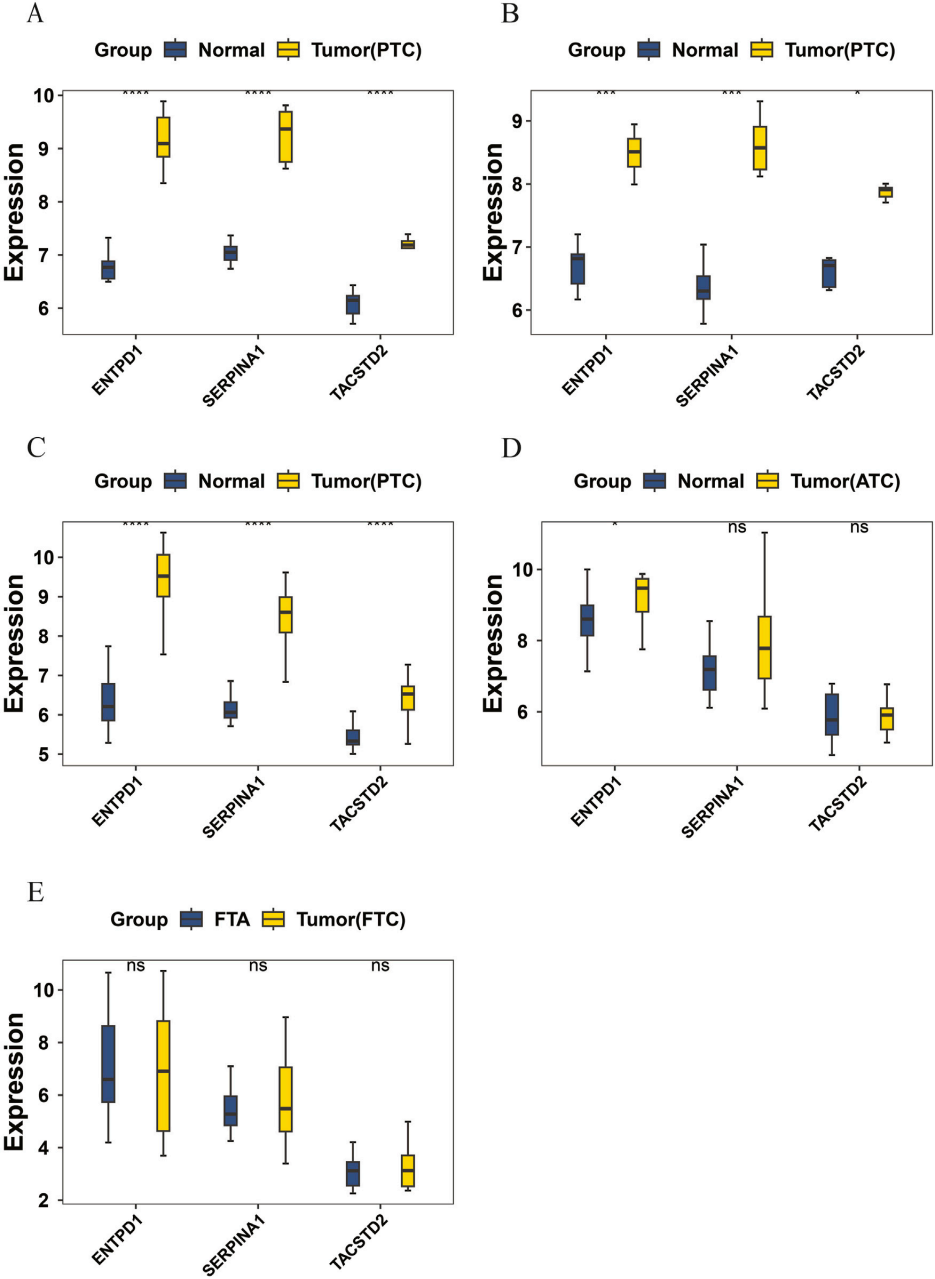
**(A)** Expression comparison of candidate biomarkers in the training set GSE3467; **(B)** expression comparison of candidate biomarkers in the validation set GSE3678; **(C)** expression comparison of candidate biomarkers in the validation set GSE33630; **(D)** expression comparison of candidate biomarkers in the validation set GSE65144; **(E)** expression comparison of candidate biomarkers in the validation set GSE82208.

Based on the aforementioned methods, the differences in candidate biomarker expressions in the tumor and normal groups were analyzed in the training set GSE3467 (Figure 3A) and validation sets GSE3678 (Figure 3B) (Supplementary Table S8) and GSE33630 (Figure 3C) (Supplementary Table S9). A *p*-value of less than 0.05 was considered statistically significant. Genes showing consistent expression trends and significant differences between groups across these three datasets were further analyzed as candidate biomarkers for PTC. The results indicated that the three candidate biomarkers showed significant differences in the tumor and normal groups in both the training and validation sets; thus, ENTPD1, SERPINA1, and TACSTD2 were all considered candidate biomarkers for PTC for further analysis. Additionally, the expressions of candidate biomarkers in the validation sets GSE65144 (Figure 3D) (Supplementary Table S10) and GSE82208 (Figure 3E) (Supplementary Table S11), which represent different types of thyroid cancer, were analyzed. The results showed that the candidate biomarker ENTPD1 had significant expression differences between the ATC groups, while SERPINA1 and TACSTD2 did not show significant expression differences in the ATC groups. No significant expression differences were observed for the candidate biomarkers between the FTC groups.

### ROC analysis results of candidate biomarkers

Based on the aforementioned methods, ROC curves were plotted for each candidate biomarker and combinations of the three candidate biomarkers in the sample data from the training set GSE3467 (Supplementary Figure S3A) and validation sets GSE3678 (Supplementary Figure S3B), GSE33630 (Supplementary Figure S3C), GSE65144 (Supplementary Figure S3D), and GSE82208 (Supplementary Figure S3E). Candidate biomarkers with AUC values greater than 0.7 in both the training set GSE3467 and validation sets GSE3678 and GSE33630 were defined as biomarkers for PTC for further analysis. The results showed that the AUC values for the three candidate biomarkers, namely, TACSTD2, ENTPD1, and SERPINA1, were all greater than 0.7 in the training set GSE3467 and validation sets GSE3678 and GSE33630, and the AUC value for the combination of the three candidate biomarkers was superior to that of each individual candidate biomarker. Therefore, TACSTD2, ENTPD1, and SERPINA1 can all be used as biomarkers for PTC for further analysis, and the combination of TACSTD2, ENTPD1, and SERPINA1 can also be used as a biomarker for PTC. Additionally, the predictive ability of the candidate biomarkers in the validation sets GSE65144 and GSE82208, which represent different types of thyroid cancer, was analyzed. The results indicated that TACSTD2, ENTPD1, and SERPINA1 have some predictive ability for other cancer types, such as ATC and FTC, but their predictive power is not as strong as it is for PTC.

### Results of nomogram construction and biomarker assessment

Based on the aforementioned methods, a nomogram model was constructed using the biomarkers TACSTD2, ENTPD1, and SERPINA1 in all samples from the training set GSE3467 (Supplementary Figure S4A). The nomogram scores each biomarker individually, with each factor corresponding to a score, and the total score is the sum of the individual scores (total points). The total score can be used to infer the likelihood of developing PTC, with higher scores indicating a higher probability of PTC. The results show that TACSTD2, ENTPD1, and SERPINA1 can reasonably assess the likelihood of developing PTC, with TACSTD2 having the highest predictive power for the likelihood of developing PTC. To evaluate the diagnostic efficacy of the nomogram, a calibration curve was plotted (Supplementary Figure S4B). The closer the curve is to the dashed line with a slope of 1, the more accurate the prediction. The results indicate that the diagnostic efficacy of the nomogram is accurate.

### Results of GSEA of biomarkers

Based on the aforementioned methods, GSEA was conducted in the training set GSE3467 to explore the potential functions of the biomarkers, and the top five enrichment results for biomarkers TACSTD2, ENTPD1, and SERPINA1 are displayed in Supplementary Figure S5A (p < 0.05, FDR <0.25, and |NES| > 1). The results indicate that TACSTD2 upregulates the valine, leucine, and isoleucine degradation and Parkinson’s disease pathways and downregulates the spliceosome, cytokine–cytokine receptor interaction, and neuroactive ligand–receptor interaction pathways; ENTPD1 upregulates the valine, leucine, and isoleucine degradation pathways and downregulates the spliceosome, Parkinson’s disease, propanoate metabolism, and cytokine–cytokine receptor interaction pathways; and SERPINA1 upregulates the Parkinson’s disease, cytokine–cytokine receptor interaction, and cell adhesion molecule (CAM) pathways and downregulates the spliceosome and neuroactive ligand–receptor interaction pathways.

Based on the aforementioned methods, the top five pathways enriched by the biomarkers were intersected, and the results indicated that there are three common pathways, namely, the spliceosome, Parkinson’s disease, and cytokine–cytokine receptor interaction pathways (Supplementary Figure S6). Additionally, “Cytoscape” was used to analyze the relationships of genes in the aforementioned three common pathways, examining the genes significantly enriched in the pathways related to the biomarkers (Supplementary Table S6). The results showed that there are 219 enriched genes across the three common pathways, with 66 genes in the spliceosome pathway, 54 genes in the Parkinson’s disease pathway, and 99 genes in the cytokine–cytokine receptor interaction pathway, with no common enriched genes among these three pathways (Supplementary Figure S7).

### Results of quality control analysis for PTC single-cell data

Based on the aforementioned methods, the analysis before and after quality control for GSE191288 was performed. Before quality control, nCount RNA (Supplementary Figure S8A), nFeature RNA (Supplementary Figure S8B), and percent.MT (Supplementary Figure S8C) were calculated, and a correlation graph between each indicator was plotted (Supplementary Figure S8D), including a total of 30,502 cells and 32,735 genes. The correlation between nFeature RNA and nCount RNA was 0.88, indicating a positive correlation, while the correlation between percent.MT and nCount RNA was −0.11, indicating a negative correlation. Cells with fewer than 200 genes, genes covered by fewer than three cells, and cells with more than 30% mitochondrial genes were filtered out. Cells with gene counts ≤ 500 and ≥ 10,000 were removed, and genes with count numbers ≤ 1000 and ≥ 10,000 were also removed. After calculating nCount RNA (Supplementary Figure S9A), nFeature RNA (Supplementary Figure S9B), and percent.MT (Supplementary Figure S9C), the quality-controlled cells and genes were used for subsequent analysis, including a total of 30,502 cells and 15,250 genes. A correlation graph between each indicator was plotted (Supplementary Figure S9D), with the correlation between nFeature RNA and nCount RNA being 0.93, indicating a positive correlation, and the correlation between percent.MT and nCount RNA being −0.16, indicating a negative correlation. The results indicate that the data quality is good and improves further after quality control.

### Results of highly variable gene identification

**FIGURE 4:**
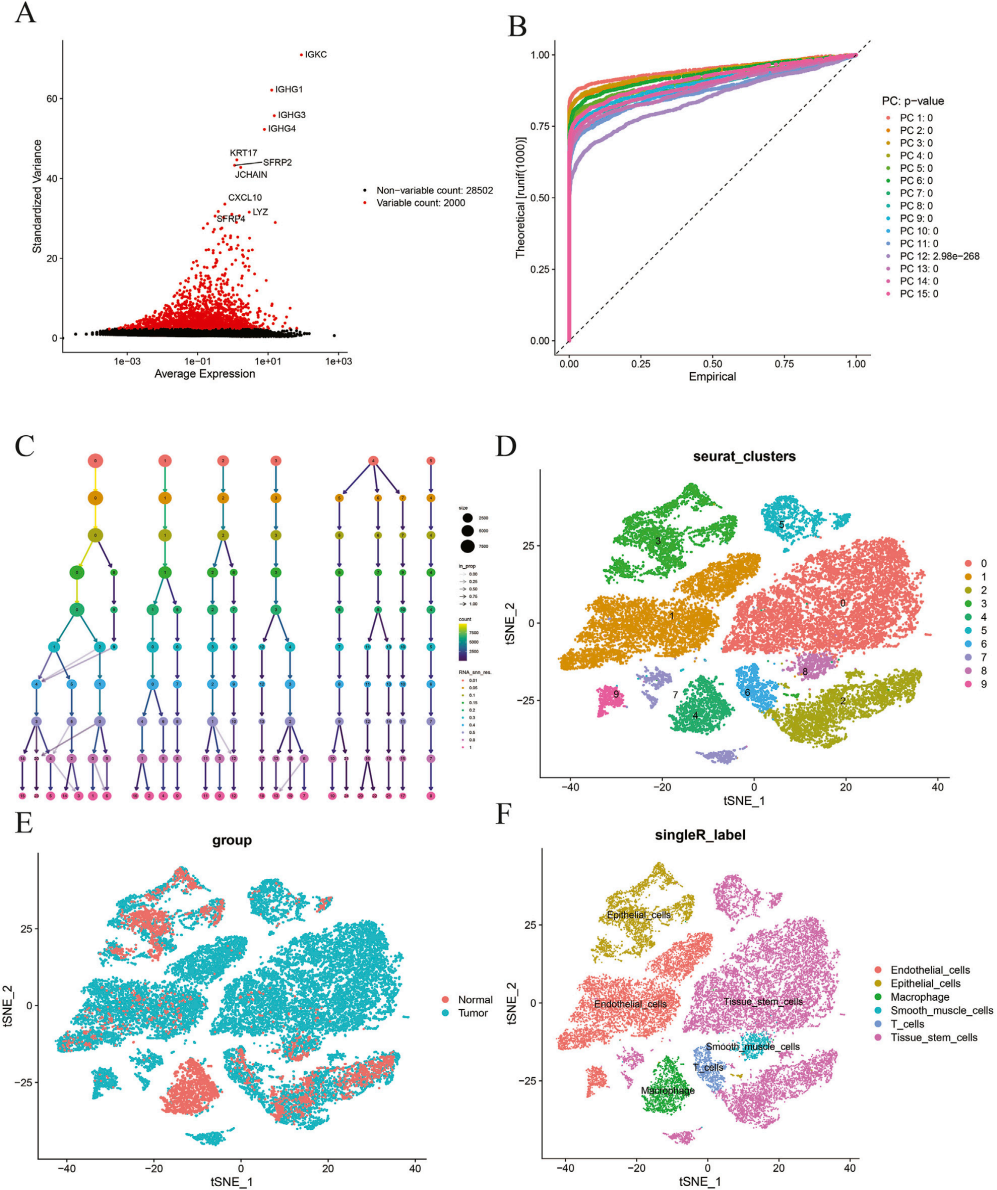
**(A)** Selection of highly variable genes; **(B)** principal component analysis; **(C)** ClusTree visualization; **(D)** t-SNE dimensionality reduction and clustering analysis at resolution = 0.15; **(E)** t-SNE dimensionality reduction and clustering analysis by group; **(F)**-SingleR cell annotation analysis.

Based on the aforementioned methods, in GSE191288, the top 2,000 highly variable genes with significant fluctuations are displayed in Figure 4A. The results show that the top 10 highly variable genes are, namely, IGKC, IGHG1, IGHG3, IGHG4, KRT17, SFRP2, JCHAIN, CXCL10, LYZ, and SFRP4.

### Results of cell dimensionality reduction and cluster annotation

Based on the aforementioned methods, in GSE191288, the top 15 results are visualized (Figure 4B). The interaction between different clusters at different resolutions is presented in Figure 4C. A resolution of 0.15 was selected for downstream analysis, resulting in a total of 10 different cell populations, and t-SNE was used to perform dimensionality reduction and clustering visualization of the cell populations according to the resolution and group (Figures 4D, E). Finally, SingleR was used for fully automated annotation of the cell populations, and a total of six different cell populations were obtained, namely, tissue stem cells, epithelial cells, T cells, endothelial cells, macrophages, and smooth muscle cells (Figure 4F).

### Results of key cell identification

**FIGURE 5:**
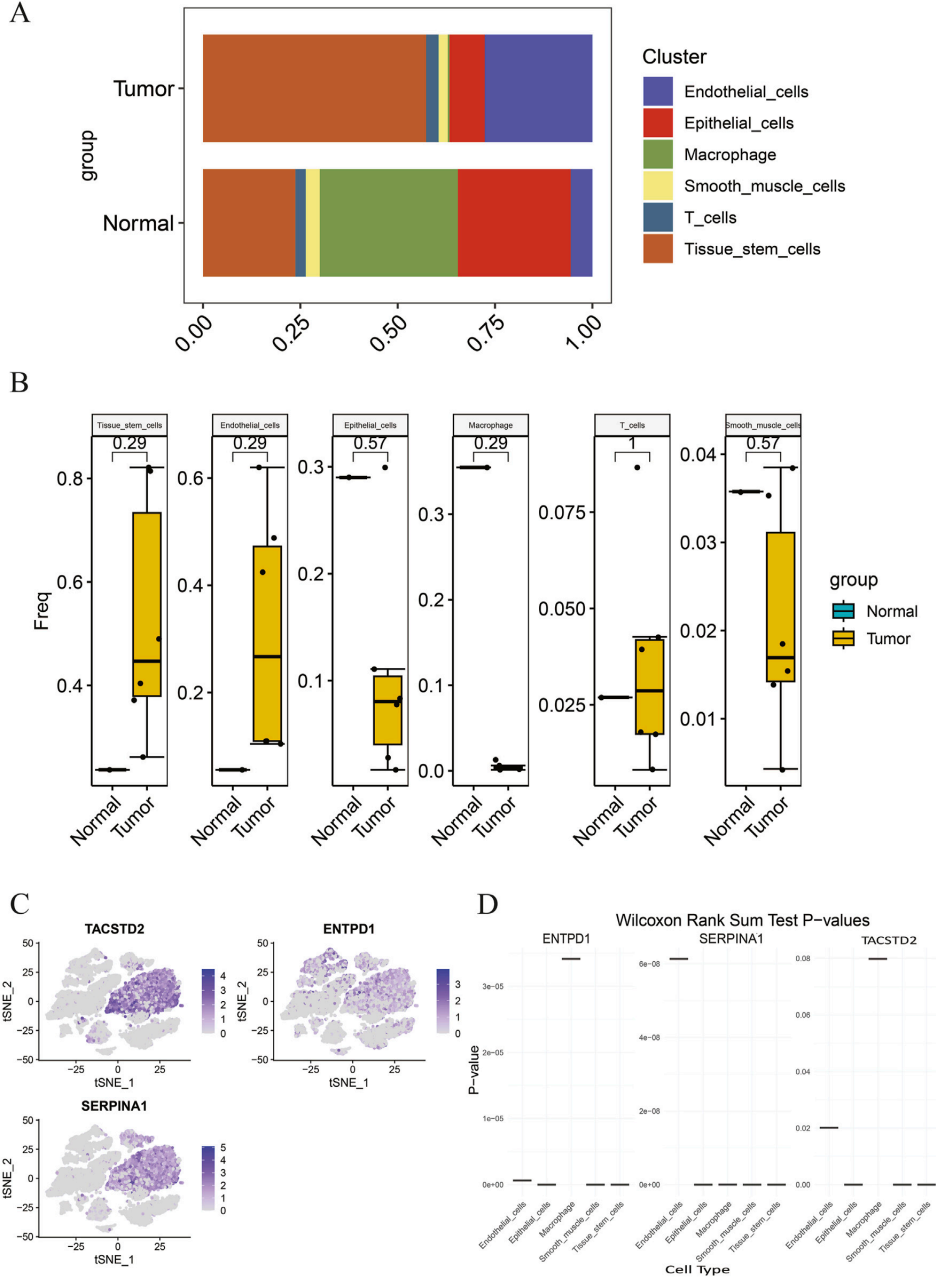
**(A)** Proportion of each cell type in different groups; **(B)** abundance differences in each cell type between different groups; **(C)** distribution of biomarkers in differential cell types; **(D)** expression of biomarkers in different cell types.

Based on the aforementioned methods, in GSE191288, the abundance of each cell type annotated above was analyzed in tumor and normal groups (Figure 5A). The differences in the abundance of each cell type between the two groups were compared and presented using box plots (Figure 5B). The results indicate that there is no change in the abundance of T cells between the tumor and normal groups. Compared to the normal group, the abundance of tissue stem cells and endothelial cells significantly increased in the tumor group, while the abundance of epithelial cells, macrophages, and smooth muscle cells significantly decreased in the tumor group. Therefore, the five cell types with increased or decreased abundance in the tumor group, namely, tissue stem cells, epithelial cells, endothelial cells, macrophages, and smooth muscle cells, were subjected to further analysis, and the distribution of the biomarkers TACSTD2, ENTPD1, and SERPINA1 in these five cell types was plotted (Figure 5C). The results show that TACSTD2 is mainly highly expressed in tissue stem cells, ENTPD1 is mainly highly expressed in tissue stem cells and epithelial cells, and SERPINA1 is mainly highly expressed in tissue stem cells. At the same time, the expression levels of each biomarker in these five cell types between the tumor and normal groups were compared. To further determine the cell types that play a key role in the tumor group, cells with significant expression differences in the biomarkers in these five cell types were defined as key cells (*p* < 0.01) (Figure 5D) (Supplementary Table S12). The results indicate that the biomarkers TACSTD2, ENTPD1, and SERPINA1 have significant expression differences in tissue stem cells, epithelial cells, and smooth muscle cells; therefore, tissue stem cells, epithelial cells, and smooth muscle cells are defined as key cells.

### Results of key cell communication analysis

**FIGURE 6:**
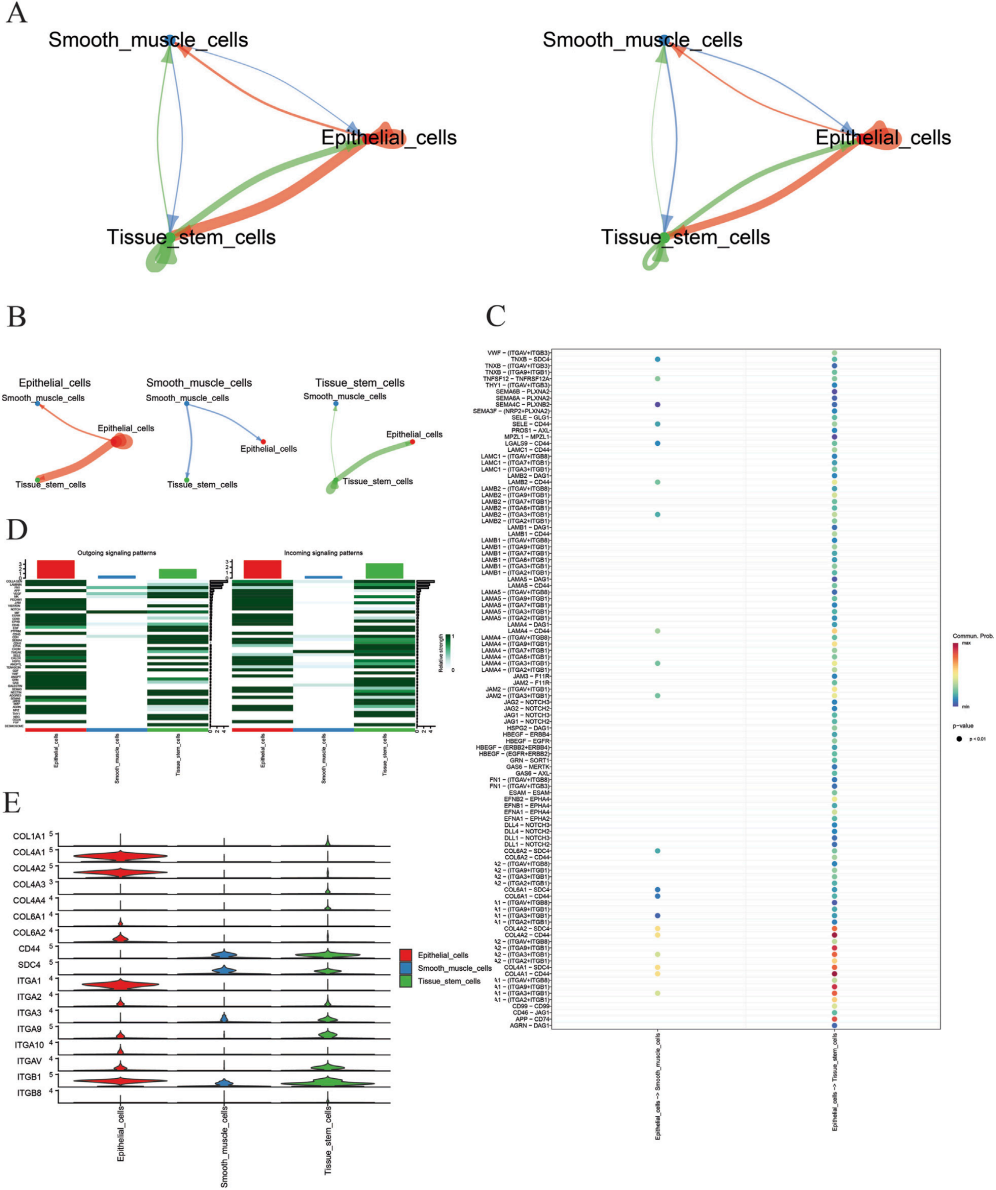
**(A)** Cell communication interaction graph; **(B)** cell sub-network communication interaction graph; **(C)** key cell receptor–ligand pairs; **(D)** signaling pathway heatmap; **(E)** violin plots of representative gene expression in signaling pathways.

Based on the aforementioned methods, in GSE191288, key cells—tissue stem cells, epithelial cells, and smooth muscle cells—were subjected to cell–cell communication analysis. The aggregated cell–cell communication network was calculated to display the quantity and strength of the cell communication network (Figure 6A) and the cell communication sub-networks (Figure 6B). The results indicate that epithelial cells have the strongest communication network quantity and strength, surpassing tissue stem cells and smooth muscle cells. Then, with epithelial cells as the source and tissue stem cells and smooth muscle cells as the targets, a bubble chart visualizes the interactions between cells through ligand–receptor pairs (Figure 6C). The results show that epithelial cells mainly interact with tissue stem cells and smooth muscle cells through the COL4A1–CD4 and COL4A2–CD4 ligand–receptor pairs. Further visualization of the signaling pathways that contribute the most to the output or input signals of tissue stem cells, epithelial cells, and smooth muscle cells is shown in Figure 6D. The results indicate that the collagen signaling pathway is the signaling pathway that contributes the most to the output or input signals of these three cell types. Finally, a violin plot is used on the collagen signaling pathway to display the expression of representative genes (Figure 6E). The results show that ligands COL4A1 and COL4A2 were highly expressed in epithelial cells, while the receptor CD4 showed elevated expression in both tissue stem cells and smooth muscle cells.

### Results of pseudotime analysis of key cells

**FIGURE 7:**
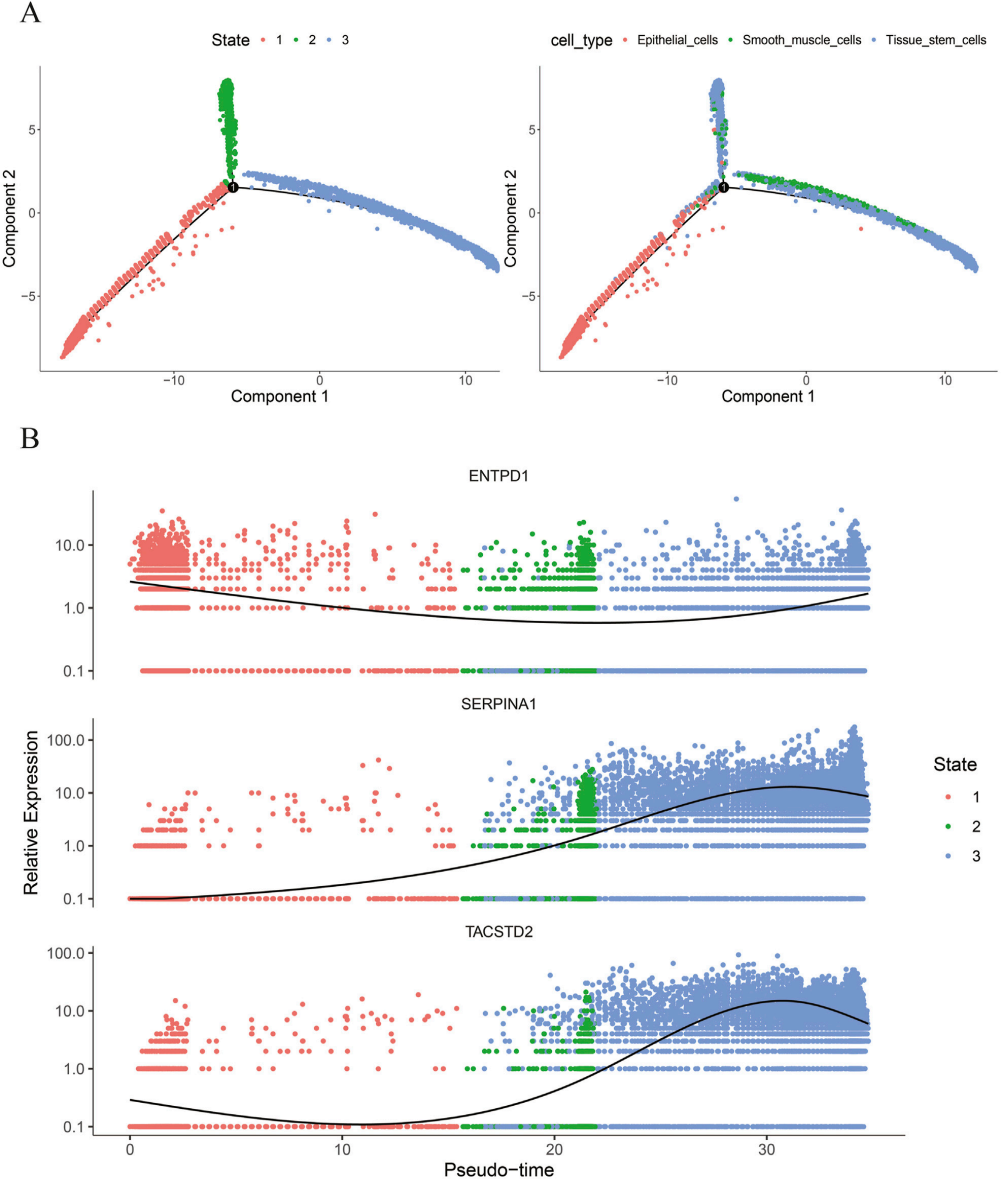
**(A)** Pseudotime analysis of key cells; **(B)** expression of biomarkers at different time points in the differentiation trajectories of key cells.

Based on the aforementioned methods, in GSE191288, pseudotime trajectories were constructed for key cells—tissue stem cells, epithelial cells, and smooth muscle cells, and pseudotime axis trajectory analysis was performed (Figure 7A); meanwhile, the expressions of biomarkers TACSTD2, ENTPD1, and SERPINA1 at different time points in the differentiation trajectories of key cells were visualized (Figure 7B). The results indicate that tissue stem cells, epithelial cells, and smooth muscle cells are involved in three stages of the cell differentiation process: epithelial cells represent the first stage, smooth muscle cells represent the second stage, and tissue stem cells represent both the second and third stages. The expression of ENTPD1 first decreases and then increases across the three different stages of differentiation; the expression of SERPINA1 first increases and then decreases across the three stages, and the expression of TACSTD2 first decreases, then increases, and finally decreases again across the three different stages of differentiation. Furthermore, ENTPD1 showed higher expression levels in the epithelial cell differentiation stage, while SERPINA1 and TACSTD2 showed higher expression levels in the tissue stem cell differentiation stage.

### Results of RT-qPCR biomarker analysis of PTC cell lines

**FIGURE 8:**
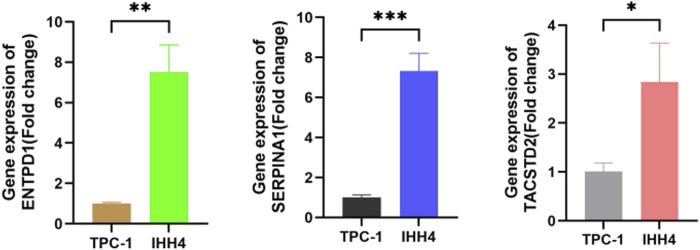
Relative mRNA expression (2^−ΔΔCt^) of biomarkers (ENTPD1, SERPINA1, and TACSTD2) normalized to TPC-1 cell.

To further validate our result, we selected biomarkers (ENTPD1, SERPINA1, and TACSTD2) for RT-qPCR analysis in PTC cell lines (TPC-1 and IHH4). Due to experimental limitations, normal thyroid cells were not included as controls in this study. Therefore, we used TPC-1 as the reference cell line to calculate relative expression differences between the two cell lines. These results were then correlated with the log2FC values derived from control vs. tumor groups in the PTC GSE3467 dataset to indirectly assess expression trends between normal and tumor conditions. The results are presented in Figure 8. RT-qPCR revealed significantly higher expression of ENTPD1, SERPINA1, and TACSTD2 in IHH4 than in TPC-1 (2^−ΔΔCt^ >1.5-fold; *P* < 0.05), and this trend aligned with PTC GSE3467 dataset, showing upregulation in PTC tumors (log2FC > 1.0) (Supplementary Table S13).

## Discussion

Thyroid cancer is the ninth most common cancer worldwide (Sung et al., 2021). In the last few years, the incidence of thyroid cancer has continuously increased, mainly because of the increased detection rate of PTC (Kitahara and Sosa, 2016). However, the carcinogenesis of PTC is a complex biological process characterized by various molecular abnormalities, and the reasons for its high prevalence remain poorly understood (Teng et al., 2018). Thus, for diagnosis and prognosis, there remains a compelling need to decode novel molecular targets and/or processes that underlie the pathogenesis and progression of PTC (Ye et al., 2017).

Our integrative analysis followed a logical workflow to first identify candidate biomarkers and key cells from RNA-seq and scRNA-seq, then validate their diagnostic values, and finally investigate their cellular functions in the PTC microenvironment. Through this systematic multi-omics approach, we first established the diagnostic values of ENTPD1, SERPINA1 and TACSTD2 and then uncovered their roles in key cellular interactions and differentiation trajectories.

In this study, we obtained PTC RNA-seq and scRNA-seq datasets from the GEO database. Through differential gene analysis, WGCNA, and MCODE analysis of the RNA-seq data, we identified 12 candidate genes. We then applied LASSO, SVM-RFE, and Boruta algorithms to screen these 12 candidate genes and performed ROC analysis and nomogram analysis, ultimately identifying three biomarkers, namely, ENTPD1, SERPINA1, and TACSTD2. A search in GEO for existing PTC RNA-seq cohort studies has not identified any cohort studies that have found ENTPD1, SERPINA1, TACSTD2, or their combinations as potential biomarkers for PTC. ENTPD1, also known as CD39, is a cell membrane protein belonging to the ecto-nucleoside triphosphate diphosphohydrolase family and is highly expressed in various malignancies, such as lymphoma (Bastid et al., 2015), multiple myeloma (Häusler et al., 2011), and renal cell carcinoma (Yang et al., 2020). Additionally, CD39 is expressed on the surface of immune cells and exosomes, ultimately affecting antitumor immunity through the dual pathways of eATP consumption and adenosine accumulation (Allard et al., 2017). It is also considered a novel biomarker of T-cell exhaustion and an immune checkpoint target for cancer therapy, with potential for certain clinical applications (Allard and AllardB, 2020). Studies have predicted that ENTPD1 is closely related to the occurrence, development, and prognosis of PTC (Fan et al., 2021). SERPINA1, a 1-antitrypsin primarily synthesized in specific cells, plays multiple roles in physiological and pathological processes such as angiogenesis, intravascular fibrinolysis, and tumor metastasis (Farshchian et al., 2011). Studies have shown that SERPINA1 is significantly upregulated in PTC (Liu et al., 2023). Experimental studies have indicated that SERPINA1 is positively correlated with the proliferation and migration abilities of PTC cells (Li et al., 2023). TACSTD2, also known as Trop2, is a transmembrane glycoprotein associated with tumor invasion and proliferation (Zaman et al., 2019). Studies have shown that Trop2 is overexpressed in various tumors, such as breast cancer, cervical cancer, and colorectal cancer, and is related to the prognosis of cancer patients (Lai et al., 2024). Experimental studies have shown that Trop2 is upregulated in PTC (Attia et al., 2024). These findings are consistent with the results of this study, indicating the reliability of the methods and results of this research.

The GSEA results suggest that ENTPD1, SERPINA1, and TACSTD2 may be involved in the occurrence and development of PTC by jointly regulating the spliceosome, Parkinson’s disease, and cytokine–cytokine receptor interaction pathways. Zhang et al. (2019) found that the cytokine–cytokine receptor interaction pathway is closely related to the occurrence and development of PTC. Lin et al. (2019) found that 46 differentially expressed immune-related genes are significantly associated with the clinical outcomes of PTC patients, and functional enrichment analysis shows that these genes are actively involved in the cytokine–cytokine receptor interaction pathway. Zhang et al. (2015) demonstrated that KEGG enrichment analysis of differential genes related to PTC includes the classic signal pathway of cytokine–cytokine receptor interactions (Zhang et al., 2015). This is consistent with the results of this study. However, the relationship between the spliceosome and Parkinson’s disease pathways and PTC has not yet been reported in any studies.

The scRNA-seq analysis identified a total of six different cell types, namely, tissue stem cells, epithelial cells, T cells, endothelial cells, macrophages, and smooth muscle cells. Xianhui Ruan conducted a comprehensive analysis of the PTC scRNA-seq dataset GSE191288, setting a resolution of 0.8, and preliminarily determined 27 cell types. By analyzing all marker genes in each cluster of the dataset to select the main cell types, 10 well-annotated cell types were ultimately selected for analysis, namely, B cells, DCs, endothelial cells, epithelial cells, macrophages, monocytes, NK cells, smooth muscle cells, T cells, tissue stem cells, and iPS cells (Ruan et al., 2023). This is consistent with the six cell types annotated in the analysis results of this study. Further analysis of the abundance of each cell type in tumor and normal group samples showed that there were significant changes in the abundance of tissue stem cells, epithelial cells, endothelial cells, macrophages, and smooth muscle cells in the tumor group. The Wilcoxon rank-sum test was used to compare the expression levels of the biomarkers ENTPD1, SERPINA1, and TACSTD2 in the cell types with significant changes, ultimately identifying tissue stem cells, epithelial cells, and smooth muscle cells as key cells in PTC.

Cell communication analysis of key cells revealed that epithelial cells mainly interact with tissue stem cells and smooth muscle cells through the ligand–receptor pairs COL4A1–CD4 and COL4A2–CD4, and violin plots demonstrated that ligands COL4A1 and COL4A2 were highly expressed in epithelial cells, while the receptor CD4 showed elevated expression in both tissue stem cells and smooth muscle cells. The collagen signaling pathway is the most important pathway; although the cytokine–cytokine receptor pathway (GSEA) and the collagen pathway (scRNA-seq) appear distinct, they are mechanistically interconnected—functional synergy. Cytokine pathways regulate collagen-modifying enzymes (e.g., LOXL2), aligning with the microenvironment remodeling mechanism (Pu et al., 2021b). This indicates that the ligand–receptor pairs COL4A1–CD4 and COL4A2–CD4 play a key role in the communication network between epithelial cells and both tissue stem cells and smooth muscle cells through the collagen signaling pathway.

Pseudotime analysis revealed that epithelial cells dominate the initial differentiation stage, suggesting that they may either represent the cellular origin of PTC or an early differentiated state. This implicates epithelial cells as the likely source of malignant transformation. In the second differentiation phase, the emergence of smooth muscle cells indicates possible epithelial–mesenchymal transition (EMT) or mesenchymal transformation within PTC, which correlates with tumor invasiveness and potential involvement in stromal remodeling processes such as collagen deposition and fibrosis. Tissue stem cells, spanning multiple pseudotime stages, exhibit bidirectional differentiation potential, likely driving tumor initiation, therapy resistance, and recurrence. Notably, ENTPD1 expression is elevated during epithelial cell differentiation, implying its regulatory role in the malignant transformation of epithelial cells. Concurrently, SERPINA1 and TACSTD2 show peak expression in tissue stem cell phases, suggesting their cooperative regulation of stem-like malignant properties in thyroid cancer. Collectively, these findings propose that ENTPD1, SERPINA1, and TACSTD2 may coordinately promote PTC progression by initiating epithelial transformation and maintaining aggressive stem-like subpopulations.

RT-qPCR analysis demonstrated significantly higher expression levels of ENTPD1, SERPINA1, and TACSTD2 in IHH4 cells than in TPC-1 cells (2^−ΔΔCt^ >1.5-fold, *P* < 0.05), suggesting that the IHH4 cell line may better recapitulate PTC subtypes with naturally elevated expression of these biomarkers and could be prioritized for subsequent experiments. Furthermore, RNA-seq data from GSE3467 (Supplementary Table S12) revealed consistent upregulation of these genes in PTC tumor tissues *versus* normal controls (log2FC > 1.0), which aligns with the elevated expression pattern observed in IHH4 cells. This concordance between our experimental data and public database results not only validates the reliability of the identified biomarkers but also supports the potential diagnostic utility of ENTPD1, SERPINA1, and TACSTD2 for PTC. The molecular characteristics preserved in IHH4 cells make them an ideal model system for functional studies of these genes. Due to experimental limitations, this study did not include normal thyroid cell controls for direct comparison, which warrants further validation using clinical specimens or primary cell cultures in future investigations.

In conclusion, this work, based on bioinformatics analysis techniques, identified three biomarkers that can predict PTC at the transcriptome and single-cell levels, with their combination demonstrating strong predictive ability for PTC. Additionally, it was found that these three biomarkers have some predictive ability for other types of thyroid cancer, such as ATC and FTC, and tissue stem cells, epithelial cells, and smooth muscle cells as key cells in PTC at the cellular level. Further elucidation of their mechanisms of action in PTC provides a new research perspective for the precision treatment of PTC in future clinical and basic research.

Compared to previous PTC biomarker studies (Zhang et al., 2023), our integrative approach combining bulk and single-cell transcriptomics provides both diagnostic markers and their cellular-level mechanisms. Although existing machine learning studies typically use one algorithm, our multi-algorithm consensus improves reliability, as evidenced by higher AUC in independent validations.

The workflow is generalizable to other cancers with appropriate dataset availability. The WGCNA–machine learning pipeline can be applied to any tumor with bulk RNA-seq data, while the single-cell analysis framework is adaptable to malignancies with tumor microenvironment complexity. However, clinical translation requires validation in prospectively collected cohorts with standardized protocols.

## Conclusion

In summary, this study combines transcriptomic analysis techniques and single-cell transcriptomic analysis techniques to identify ENTPD1, SERPINA1, and TACSTD2 as potential biomarkers for PTC at the transcriptomic level. The cytokine–cytokine receptor interaction signaling pathway is closely associated with the occurrence and development of PTC, and tissue stem cells, epithelial cells, and smooth muscle cells are considered key cells in PTC at the cellular level. The ligand–receptor pairs COL4A1–CD4 and COL4A2–CD4 play a crucial role in the collagen signaling pathway, mediating the communication network between epithelial cells, tissue stem cells, and smooth muscle cells, and ENTPD1, SERPINA1, and TACSTD2 play key roles in the differentiation process of the three key cell types, namely, tissue stem cells, epithelial cells, and smooth muscle cells. These findings may contribute to the development of early diagnostic strategies, prognostic markers, and therapeutic targets for PTC.

## Data Availability

The original contributions presented in the study are included in the article/Supplementary Material; further inquiries can be directed to the corresponding author.
