# Supplementary material for: Integrative transcriptomics and single-cell transcriptomics analyses reveal potential biomarkers and mechanisms of action in papillary thyroid carcinoma
Source: Front Genet. 2025 May 30;16:1536198. doi: 10.3389/fgene.2025.1536198 (PMC12162626; doi:10.3389/fgene.2025.1536198)
Supplement: Supplementary file 1 [file Supplementaryfile1.docx]

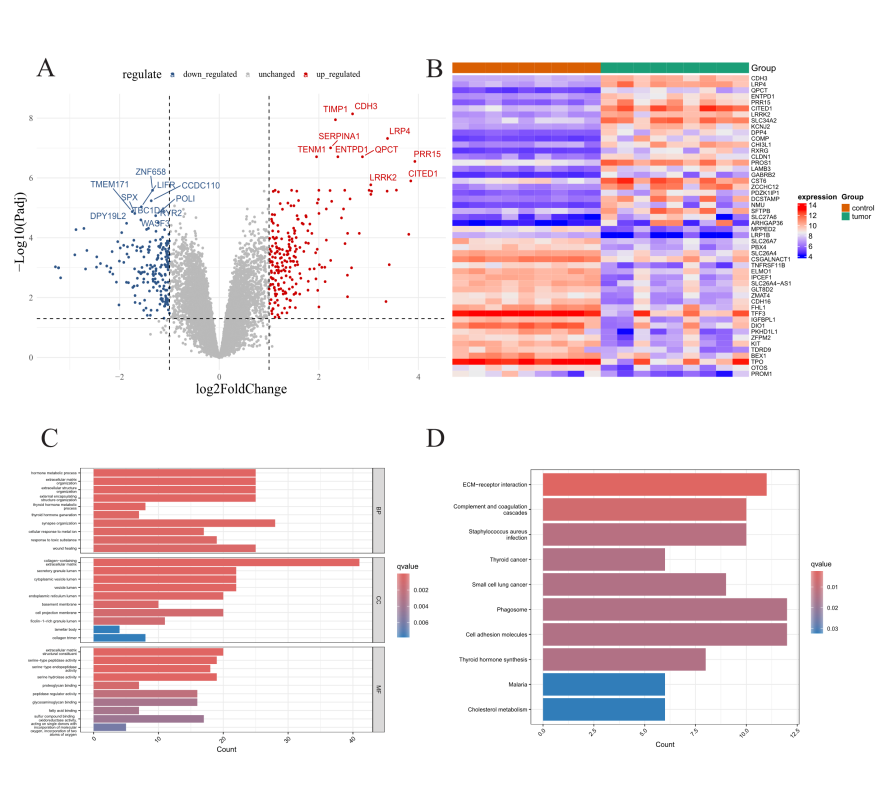


Supplementary Figure 1 A-Volcano plot of differential genes; B-Heatmap of differential genes; C-GO enrichment analysis of differential genes; D-KEGG enrichment analysis of differential genes


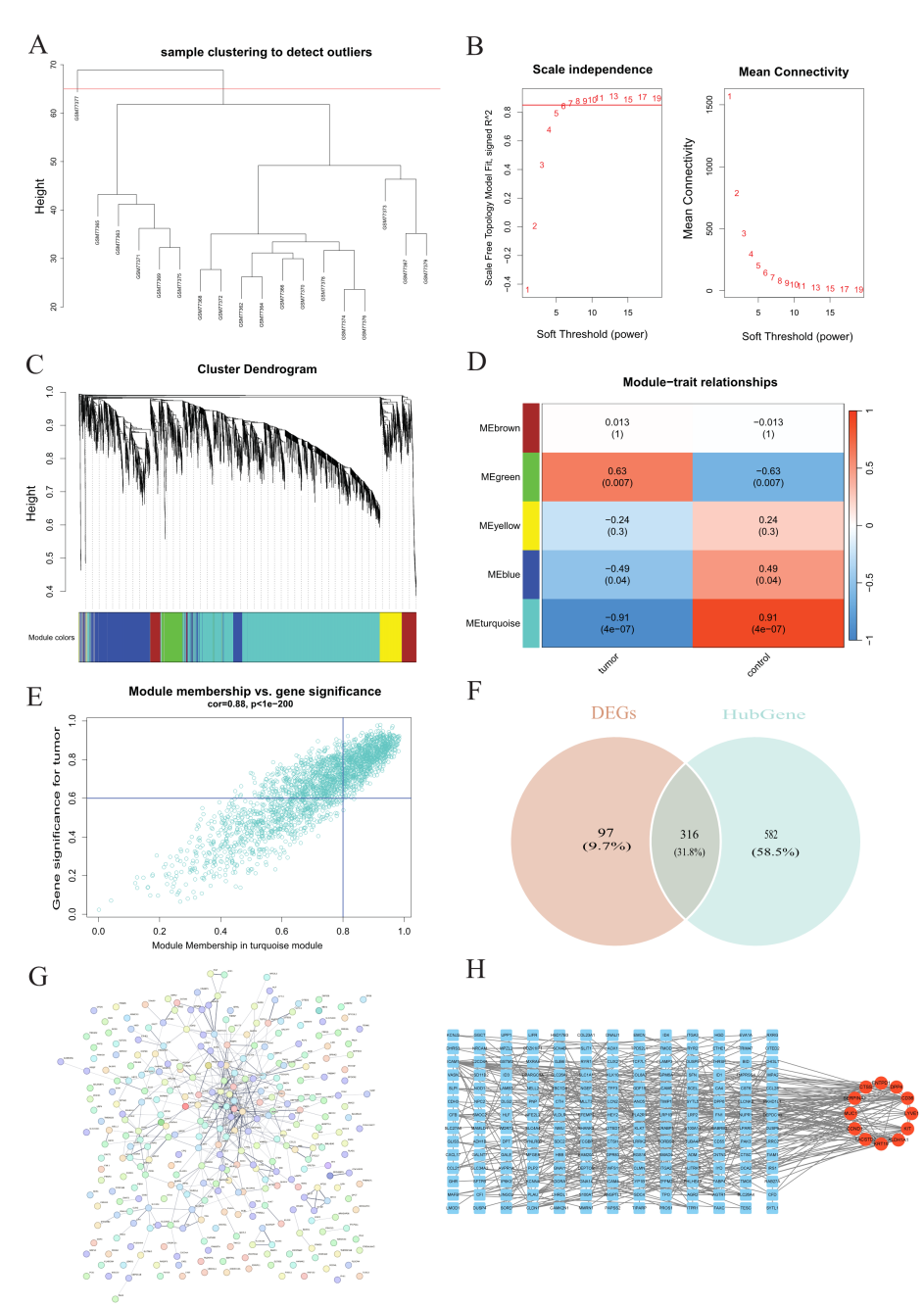


Supplementary Figure 2 A-Hierarchical Clustering Analysis; B-Determination of the Soft Threshold;C-Gene Clustering Dendrogram; D-Heatmap of Correlation between Modules and Group Scores; E-Scatter Plot of MM and GS Correlation; F-Selection of DEGs-HubGene-Intersecting Genes; G-PPI Network Interaction of Intersecting Genes; H-Identification of Candidate Genes (Orange represents the 12 core genes identified by MCODE).


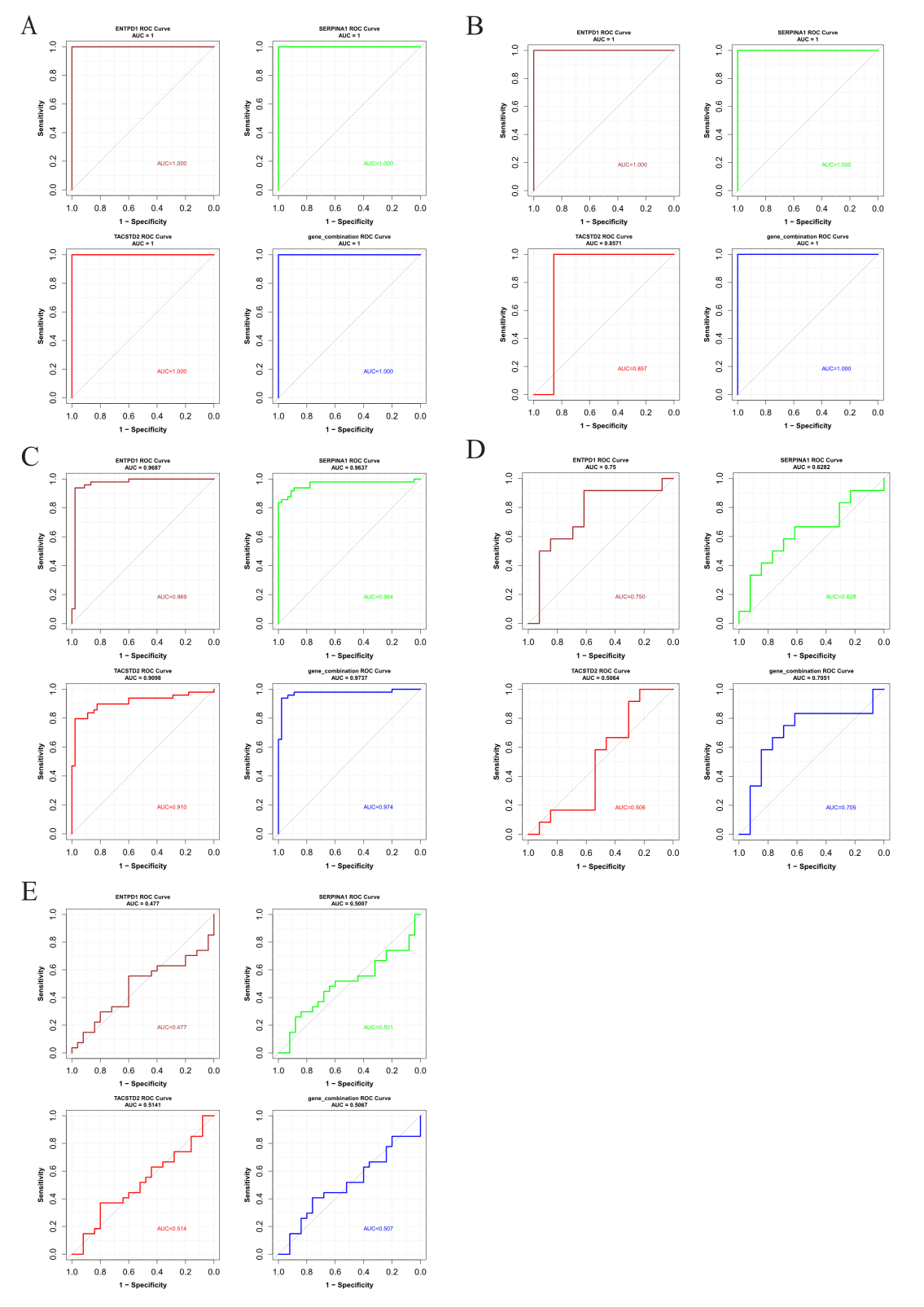


Supplementary Figure 3 A-ROC analysis of candidate biomarkers in the training set GSE3467; B-ROC analysis of candidate biomarkers in the validation set GSE3678; C-ROC analysis of candidate biomarkers in the validation set GSE33630; D-ROC analysis of candidate biomarkers in the validation set GSE65144; E-ROC analysis of candidate biomarkers in the validation set GSE82208


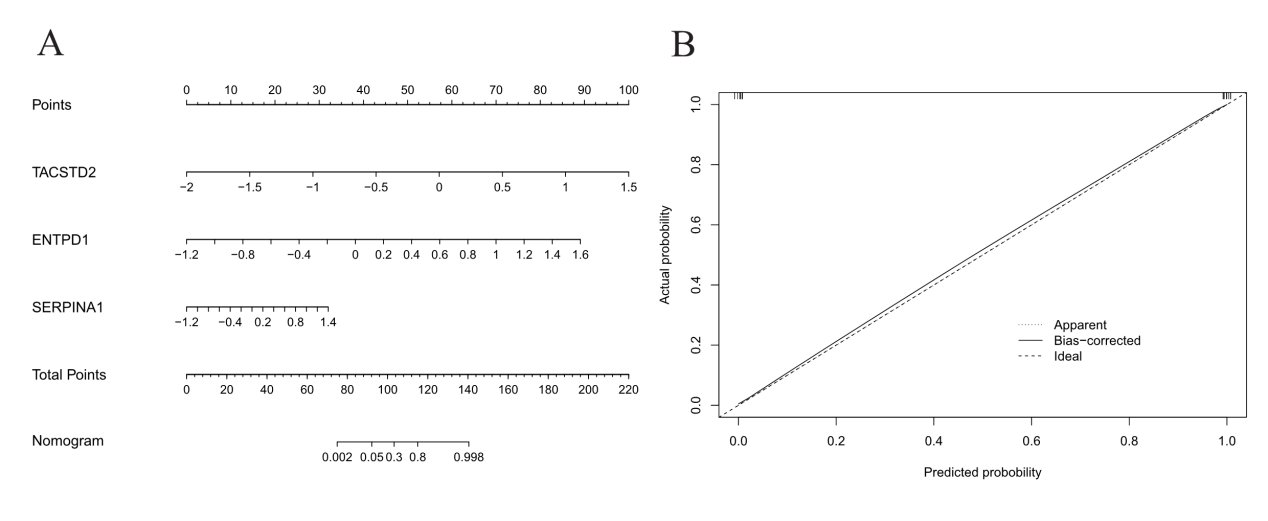


Supplementary Figure 4 A-Nomogram of biomarkers in the training set GSE3467; B-Calibration curve of candidate biomarkers in the training set GSE3467


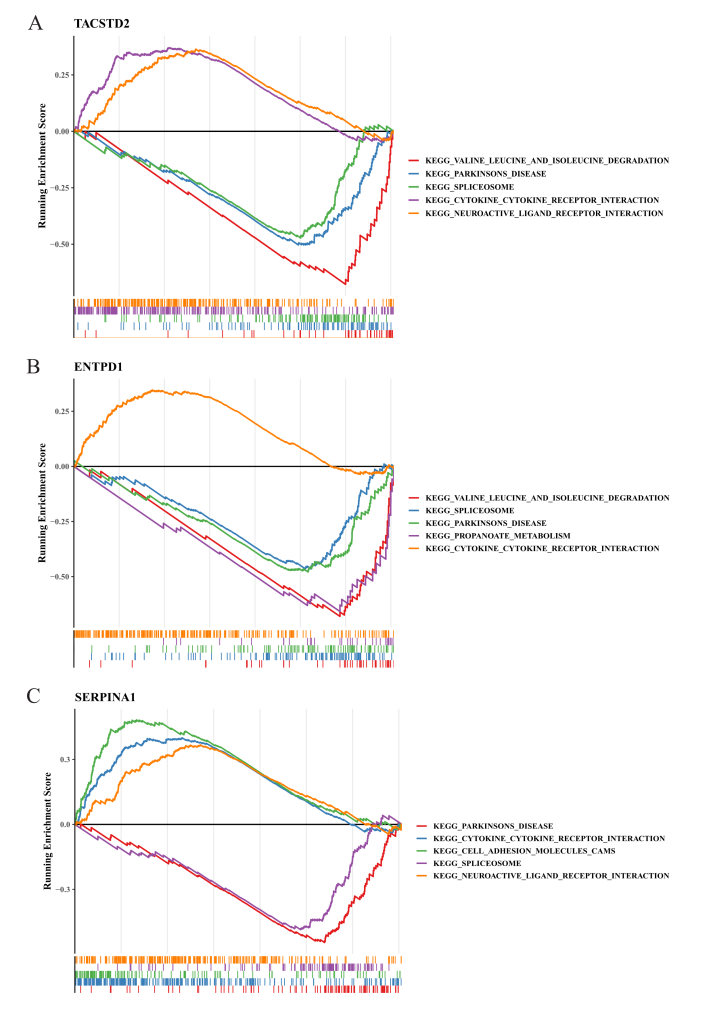


Supplementary Figure 5 A-GSEA analysis of biomarker TACSTD2; B-GSEA analysis of biomarker ENTPD1; C-GSEA analysis of biomarker SERPINA1


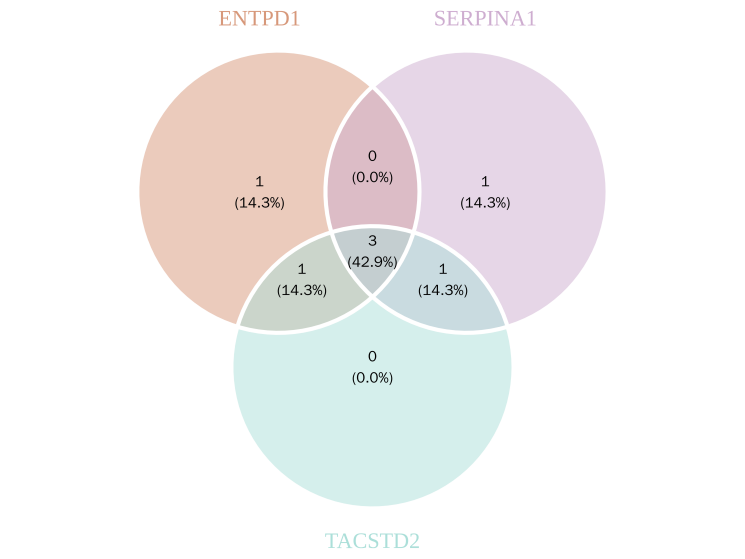


Supplementary Figure 6 Venn Diagram of Common Pathways from GSEA for Biomarkers
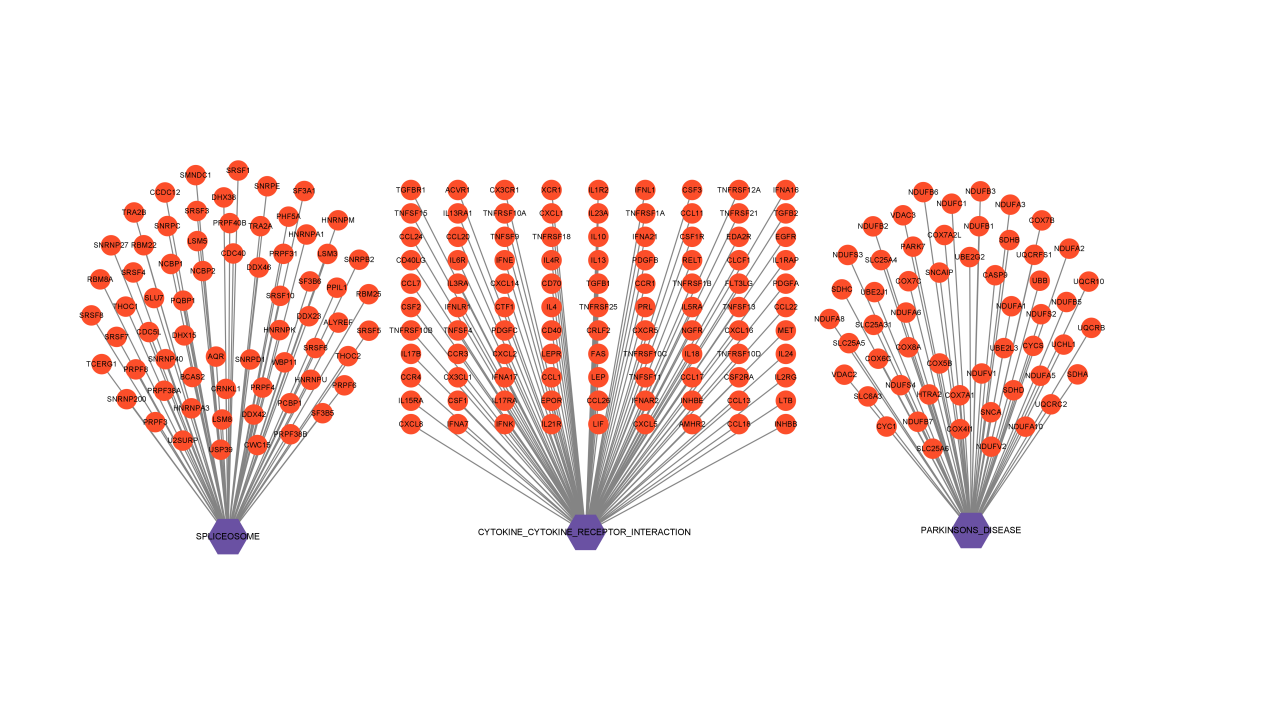


Supplementary Figure 7 Gene Analysis of Common Pathways from GSEA for Biomarkers


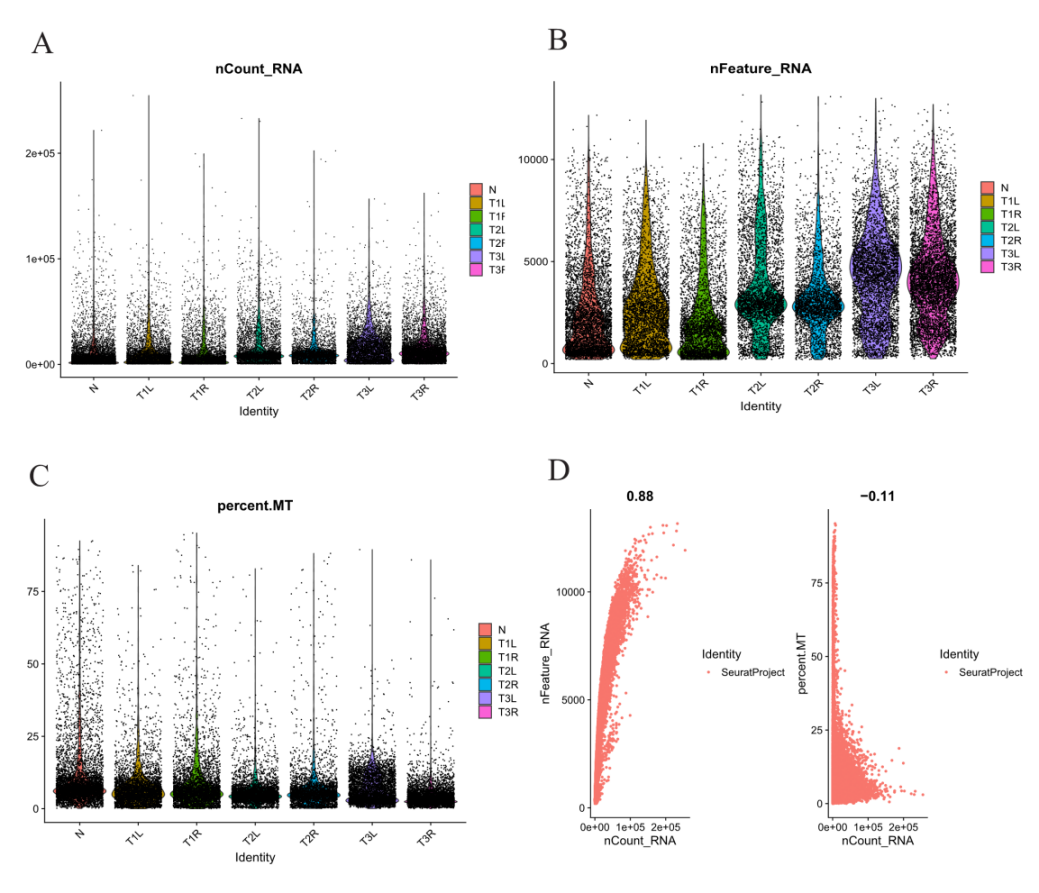


Supplementary Figure 8 A-Distribution of nFeature RNA before quality control; B-Distribution of nCount RNA before quality control; C-Distribution of percent.mt before quality control; D-Correlation of each indicator before quality control


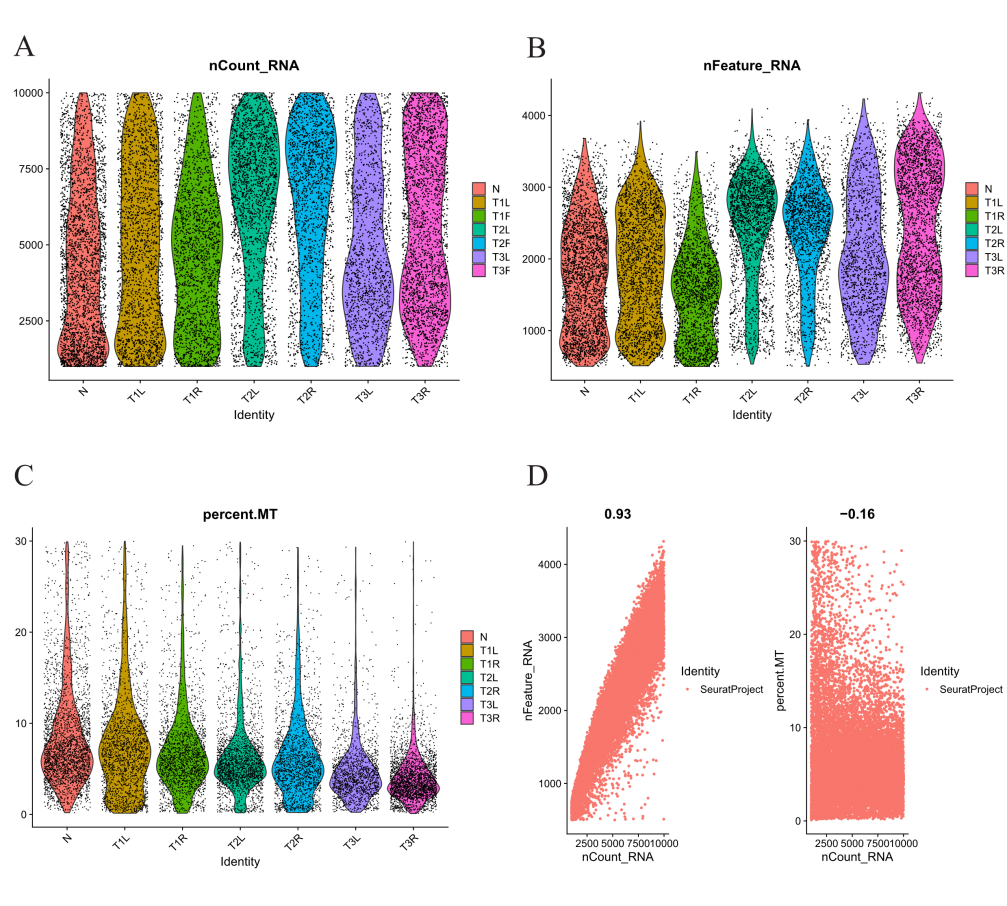


Supplementary Figure 9 A-Distribution of nFeature RNA after quality control; B-Distribution of nCount RNA after quality control; C-Distribution of percent.mt after quality control; D-Correlation of each indicator after quality control
